# Operator Auditory Perception and Spectral Quantification of Umbilical Artery Doppler Ultrasound Signals

**DOI:** 10.1371/journal.pone.0064033

**Published:** 2013-05-20

**Authors:** Ann Thuring, K. Jonas Brännström, Maria Ewerlöf, Edgar Hernandez-Andrade, David Ley, Göran Lingman, Karina Liuba, Karel Maršál, Tomas Jansson

**Affiliations:** 1 Department of Obstetrics and Gynecology, Clinical Sciences Lund, Lund University, Lund, Sweden; 2 Department of Logopedics, Phoniatrics & Audiology, Lund University, Lund, Sweden; 3 Department of Biomedical Engineering, Clinical Sciences Lund, Lund University, Lund, Sweden; 4 Perinatology Research Branch, National Institute of Child Health and Human Development, National Institutes of Health, Wayne State University, Detroit, Michigan, United States of America; 5 Department of Pediatrics, Clinical Sciences Lund, Lund University, Lund, Sweden; University of Vermont College of Medicine, United States of America

## Abstract

**Objective:**

An experienced sonographer can by listening to the Doppler audio signals perceive various timbres that distinguish different types of umbilical artery flow despite an unchanged pulsatility index (PI). Our aim was to develop an objective measure of the Doppler audio signals recorded from fetoplacental circulation in a sheep model.

**Methods:**

Various degrees of pathological flow velocity waveforms in the umbilical artery, similar to those in human complicated pregnancies, were induced by microsphere embolization of the placental bed (embolization model, 7 lamb fetuses, 370 Doppler recordings) or by fetal hemodilution (anemia model, 4 lamb fetuses, 184 recordings). A subjective 11-step operator auditory scale (OAS) was related to conventional Doppler parameters, PI and time average mean velocity (TAM), and to sound frequency analysis of Doppler signals (sound frequency with the maximum energy content [MAX_peak_] and frequency band at maximum level minus 15 dB [MAX_peak-15 dB_] over several heart cycles).

**Results:**

We found a negative correlation between the OAS and PI: median Rho −0.73 (range −0.35– −0.94) and −0.68 (range −0.57– −0.78) in the two lamb models, respectively. There was a positive correlation between OAS and TAM in both models: median Rho 0.80 (range 0.58–0.95) and 0.90 (range 0.78–0.95), respectively. A strong correlation was found between TAM and the results of sound spectrum analysis; in the embolization model the median r was 0.91 (range 0.88–0.97) for MAX_peak_ and 0.91 (range 0.82–0.98) for MAX_peak-15 dB_. In the anemia model, the corresponding values were 0.92 (range 0.78–0.96) and 0.96 (range 0.89–0.98), respectively.

**Conclusion:**

Audio-spectrum analysis reflects the subjective perception of Doppler sound signals in the umbilical artery and has a strong correlation to TAM-velocity. This information might be of importance for clinical management of complicated pregnancies as an addition to conventional Doppler parameters.

## Introduction

Doppler ultrasonography is routinely used in the surveillance of high-risk pregnancies to identify growth-restricted fetuses with impaired placental circulation and to detect imminent fetal asphyxia [1]. Blood velocity waveforms recorded from the umbilical artery reflect the placental resistance, which is related to fetal well-being. Pulsatility index (PI), derived from the velocity envelope of the Doppler spectrum, is one of the standard methods to characterize the flow velocity waveform [2], [3]. However, according to our experience, by listening to the Doppler signals from the umbilical artery, an experienced sonographer might be able to further differentiate between the Doppler audio-signals recorded from umbilical arteries of two fetuses with identical PI values, but still different clinical outcome. Additional information provided by the auditory information might be of importance for clinical management, e.g. by better timing of delivery. In fetuses with intrauterine growth restriction in the third trimester of pregnancy, delivery is recommended if the circulation in the umbilical artery shows high resistance with absent or reverse end-diastolic flow [1]. However, in fetuses with positive end-diastolic flow and increased PI, the timing of delivery is not always obvious and more data on hemodynamics in the umbilical circulation might be useful.

The quality and reliability of fetal Doppler examinations is a matter of experience and skills in optimizing the recording. This includes recognizing the timbre of the sound. To enable a wider application of the auditory control of Doppler signal, we tried to develop an objective measure of the audio signals. In two experimental fetal lamb models, we have investigated whether analysis of Doppler audio signals would provide additional information on blood circulation in the umbilical artery. Specifically, we have studied how the subjective auditory classification and sound frequency analysis of Doppler sound signals relate to conventional Doppler parameters, PI and time average mean velocity (TAM).

## Materials and Methods

### Experimental Fetal Lamb Models

The experimental studies were approved by the Animal Ethics Research Committee at the Lund University (protocols M 187-04 and M 189-07).

Two experimental models employing lamb fetuses were used for induction of blood flow changes in the umbilical artery similar to those observed in human fetuses with intrauterine asphyxia.

#### Model I: Increased vascular resistance by embolization of the placental circulation

Eight pregnant ewes with nine lamb fetuses were studied. All ewes were near term with the median gestational age 135 days (range 133–137). The mean birth weight of lambs was 4.2 kg (range 2.4–5.0). One lamb fetus was used for establishing the experimental model and set-up. Another fetus was excluded due to acidemia during the control period. The study group thus consisted of seven lamb fetuses.

The pregnant ewes were intubated after thiopental induction of anesthesia, subsequently maintained by isoflurane supplemented by **ketamine** infusion. A midline laparotomy was performed, the uterus was palpated, the hind leg of the fetus localized and exposed through a small uterotomy. A small skin incision was made in the groin of the fetus, the femoral artery was exposed and a polyvinyl catheter inserted into the descending aorta to a distance of 10 cm. The catheter was used for monitoring arterial blood pressure, obtaining blood samples, and for infusion of microspheres. Vascular resistance in the placenta was increased by stepwise embolization through injection of 0.5 ml Sephadex G-25 microspheres (12.5 mg/ml) in the fetal descending aorta every 10 min. Doppler velocimetry in the umbilical artery was performed before and immediately after each microsphere injection. Blood samples for fetal arterial pH and blood gases were taken before the first injection of microspheres and then repeatedly during the experiment.

#### Model II: Hyperdynamic circulation by inducing fetal anemia

Three pregnant ewes with six lamb fetuses were included. One fetus was excluded due to severe **intraoperative** bleeding and another one due to acidemia before the start of experimental procedure. In the remaining four fetuses, all near term (median gestational age 137 days; range 136–138) intrauterine anemia was induced.

The surgical procedure was similar to that in the embolization model except that the neck was exposed and arterial catheter was inserted directly in the carotid artery. The catheter was used for monitoring of arterial blood pressure, withdrawal of blood and blood sampling. In addition, catheterization of the ipsilateral jugular vein was done. The vein catheter was used for infusion of Ringer’s acetate solution and 6% dextran solution in proportion 1∶1. Withdrawal of 20 ml fetal arterial blood and simultaneous infusion of a corresponding fluid volume in the vein were done to induce fetal anemia. During the infusion and 5 min after, several recordings of umbilical artery Doppler signals were performed. The procedure was repeated every fifteen minutes.

In both models, the experiments continued until asystole of the fetus. After asystole, the lamb fetus was delivered by cesarean section and terminated with an intracardiac injection of potassium chloride. The uterus and abdomen of the ewe were closed and the ewe transferred to the recovery room.

### Doppler Ultrasound Recording

**Figure 1:**
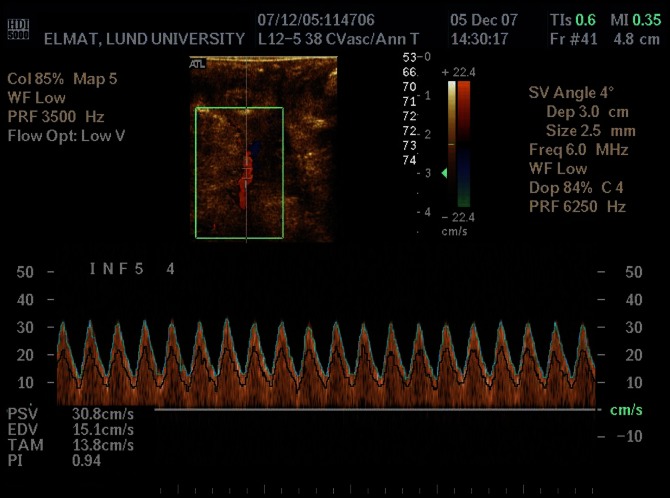
Doppler signals of blood velocity recorded from the umbilical artery of a fetal lamb. Within the Doppler spectrum displayed on the screen of the ultrasound scanner, the on-line estimated maximum velocity and the mean weighted velocity are displayed as a light blue and a black line, respectively. The automatically calculated pulsatility index (PI) and time-average velocity (TAM) values are shown in the lower left corner. The waveform of the blood velocities with positive signals throughout the heart cycle indicate a low resistance to flow in the placenta, i.e. a normal fetoplacental blood flow.

Doppler recordings of the umbilical artery flow signals were performed using a Philips HDI 5000 ultrasound system (Philips Medical Systems, Bothell, WA) with a linear 12–5 MHz transducer. The transmitted Doppler ultrasound frequency was 6 MHz. The transducer was placed directly on the wall of the uterus and the signals were recorded from the mid-portion of the umbilical cord. Pulsed-wave Doppler ultrasound supported by directional color Doppler was used, the angle of insonation always set to be as low as possible, never exceeding 30°. The sample volume size was chosen to cover the entire vessel cross-section and the high pass filter was set to be as low as possible. The PI was calculated automatically by the ultrasound machine and displayed on the screen **(** **Fig. 1** **)**. Doppler spectra were stored both on the hard disk of the ultrasound equipment and on a digital video recorder together with Doppler audio-signals for further off-line analysis.

In total, 370 Doppler recordings with video and audio signals were stored and analyzed in the embolization model, and 184 in the anemia model. The number of recordings in the individual fetuses ranged 34–88 and 32–72 in the embolization and anemia models, respectively. Each recording with optimal quality lasted for 3–8 s. The Doppler-audio information was extracted and saved as wav-files at a sampling rate of 44,100 Hz.

### Operator Auditory Classification

One experienced operator (AT) graded the audio Doppler signals on a scale from one to eleven, one representing the most abnormal and eleven the normal signal. Subsequently, the same operator evaluated once more all audio signals, this time simultaneously observing the graphic display of Doppler spectra (the sonogram). The same scale (1 to 11) was used for grading the signals. The evaluation of the audio recordings and spectra was performed blindly and in random order.

### Calculation of Time Average Mean Velocity (TAM)

For digital analysis of the recorded audio signals, successive power density spectra (PDS) in the form of a sonogram were calculated. Welch’s method [4] was used to reduce the amplitude variability of the spectrum: The signal was divided into smaller segments of 2,048 samples each, with an overlap of 50%. Each segment was windowed by a Hanning window and then fast Fourier transformed. Successive spectra were displayed as a sonogram with a scale in Hz. The following steps were further employed to convert the scale to velocity. As the maximum velocity that can be detected by a pulsed Doppler system, *v*_max_, relates to half the pulse repetition frequency, *f_PRF_*, the following relation holds for the smallest velocity increment, *v*_min_, and the smallest frequency increment (*i.e.* the frequency bin size of the sonogram *f_bin_*, here 44,100/2,048 Hz):
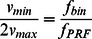



With the combination of the expression for maximum velocity in a pulsed Doppler system [5]

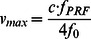
where *c* is the sound velocity in tissue, 1,540 m/s [6], and *f_0_* is the employed Doppler ultrasound frequency, here 6 MHz, the factor *k* needed to multiply each frequency was found as



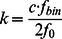



The consistency of the velocity scale was ensured by comparing peak systolic velocities presented by the ultrasound equipment and those derived from the audio signal.

The average velocity values for each segment were calculated from the sampled audio files as the ratio between the first and zeroth moment of the PDS of each 43 ms interval up to the peak velocity. This velocity was found from the maximum detectable frequency from the Fourier transform of each PDS using the modified geometric method [7]. The final value of TAM velocity was the mean value of the ratio over an integer number of heart beats.

### Sound Spectrum Analysis

The sound spectrum analysis of the audio files was done using the computer software Adobe Audition (version 3.0) (Adobe Systems Inc., San Jose, CA). Only time windows encompassing complete heart cycles were analyzed, *i.e.* windows from the systolic onset of the first complete cycle to the end diastolic velocity of the last complete cycle. To be included in the analysis, at least two complete heart cycles were required. A Fast Fourier Transform (FFT) was performed on the spectrum using a Hanning window with a 2,048 step size. The FFTs were analyzed in the frequency range 150 Hz (limit of the earphones used in clinical setting by the sonographer) to 6,008 Hz (frequency of the band stop of the recording). Two auditory prominent features were identified within this frequency range: the frequency band (in Hz) with the maximum energy content (MAX_peak_) and the high frequency cut-off (MAX_peak-15 dB_) [8]. The high-frequency cut-off was defined as the frequency band at which the energy level had decreased by 15 dB from its maximum level.

Pearson’s correlation coefficient and Spearman’s coefficient of rank correlation were used for statistical analyses as appropriate. The MedCalc version 9.1.0.1 statistical package (MedCalc software, Mariakerke, Belgium) was used for calculations.

## Results

**Figure 2:**
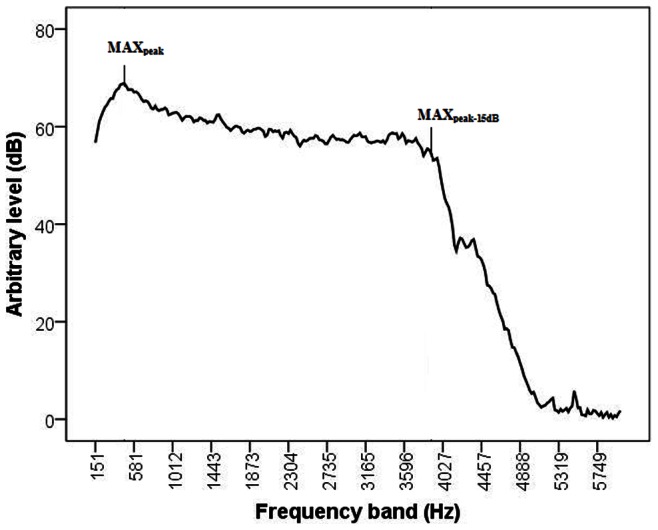
Sound spectrum analysis in a recording from a fetus with normal umbilical artery blood flow. The MAX_peak_ and MAX_peak-15 dB_ are indicated in the displayed frequencies. The operator classified this recording as “grade 11” on the scale extending from 1 (most abnormal) to 11 (normal signal).

Figures **2** **and** **3** show examples of the individual auditory analyses of Doppler sound signals recorded from fetuses with normal and severely abnormal umbilical artery blood flow, respectively. The operator classified these recordings as “grade 11” and “grade 3”, respectively, and the corresponding TAM values were 17.0 cm/s and 3.5 cm/s.

**Figure 3:**
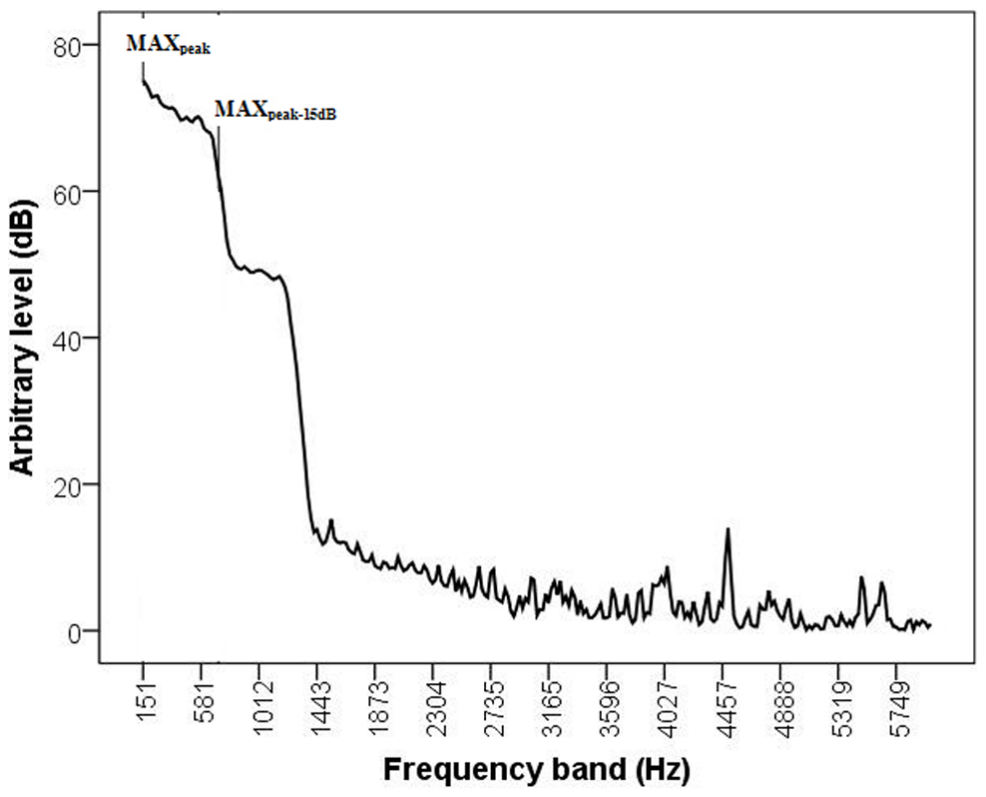
Sound spectrum analysis in a recording from a fetus with severely abnormal umbilical artery blood flow. The MAX_peak_ and MAX_peak-15 dB_ are indicated in the displayed frequencies. The operator classified this recording as “grade 3” on the scale extending from 1 (most abnormal) to 11 (normal signal).

**Figure 4:**
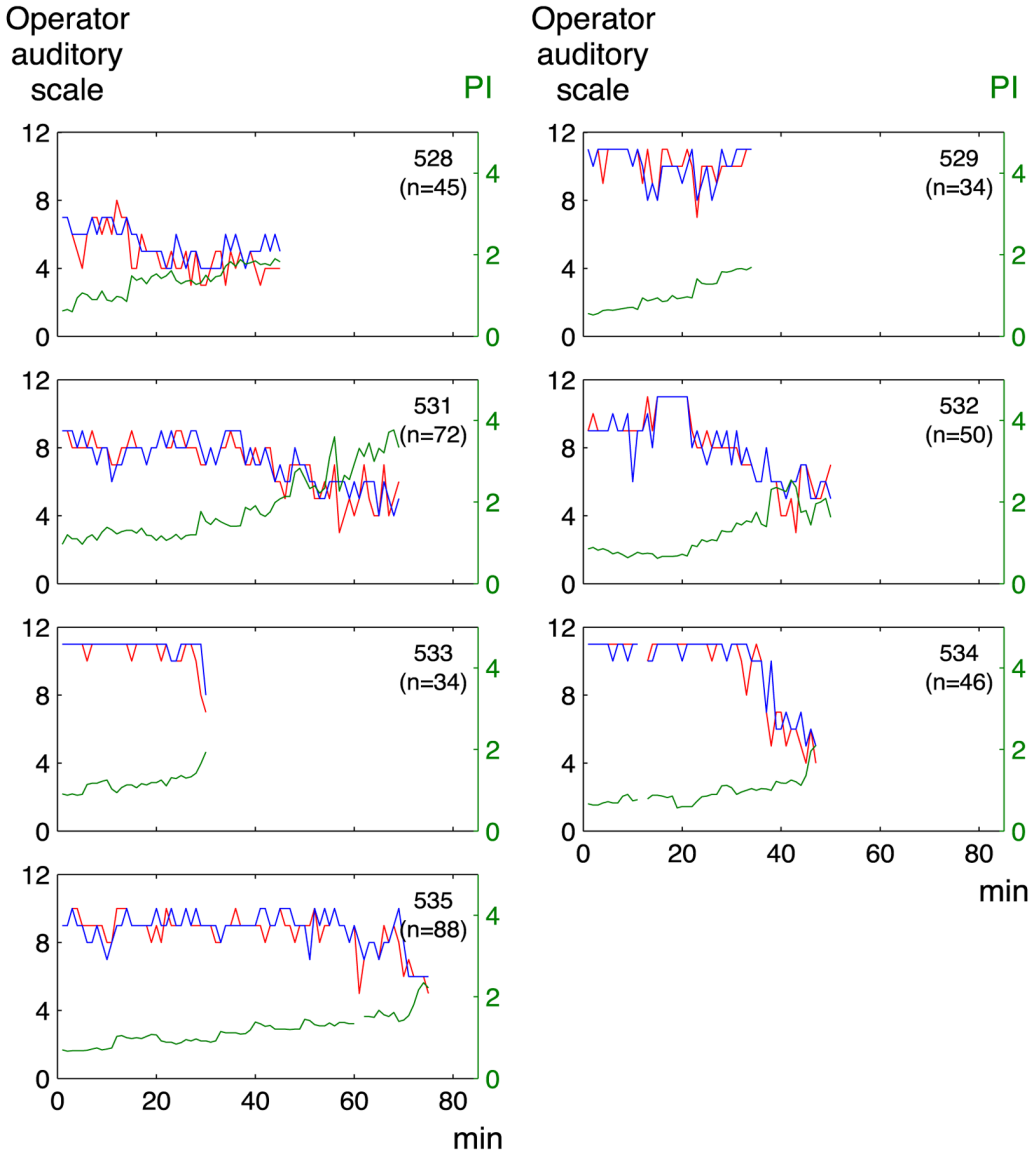
Operator auditory scale and pulsatility index for individual Doppler recordings in the embolization model. Green line: pulsatility index (PI), based on the conventional analysis of Doppler spectrum (right y-axis); red line: sonographer’s auditory perception; blue line: auditory perception supported by visual presentation of the spectrum (left y-axis); n: number of observations per lamb.

There was a significant correlation between the graded classification by the sonographer’s auditory perception and that by auditory perception supported by visual presentation of the spectrum (median Rho 0.76, range of the coefficients for individual fetuses 0.49–0.89 and median Rho 0.87, range 0.76–0.90, for the embolization and the anemia models, respectively). The agreement between the two subjective operator analyses is also illustrated in figures **4** **,** **5** **,** **6** **,** **7**.

**Figure 5:**
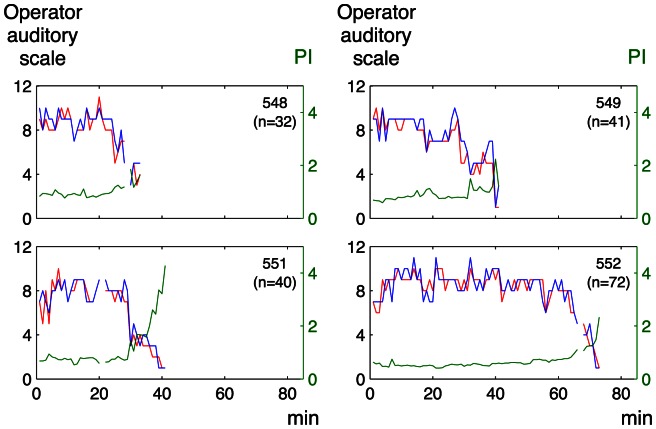
Operator auditory scale and pulsatility index for individual Doppler recordings in the anemia model. For explanations see the legend of figure 4.

**Figure 6:**
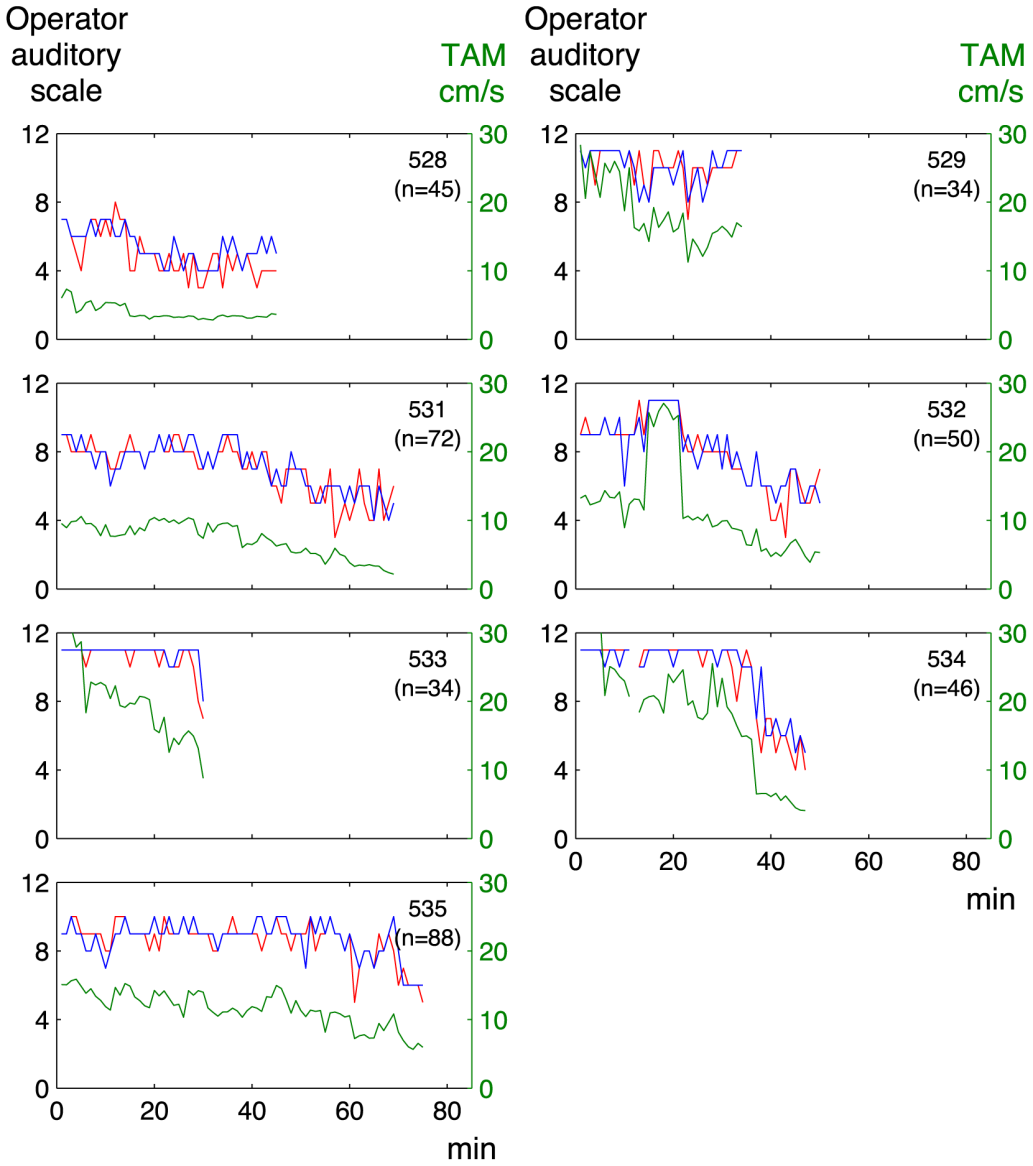
Operator auditory scale and time average mean velocity for individual Doppler recordings in the embolization model. Green line: time average mean velocity (TAM) (right y-axis); red line: sonographer’s auditory perception; blue line: auditory perception supported by visual presentation of the spectrum (left y-axis); n: number of observations per lamb.

**Figure 7:**
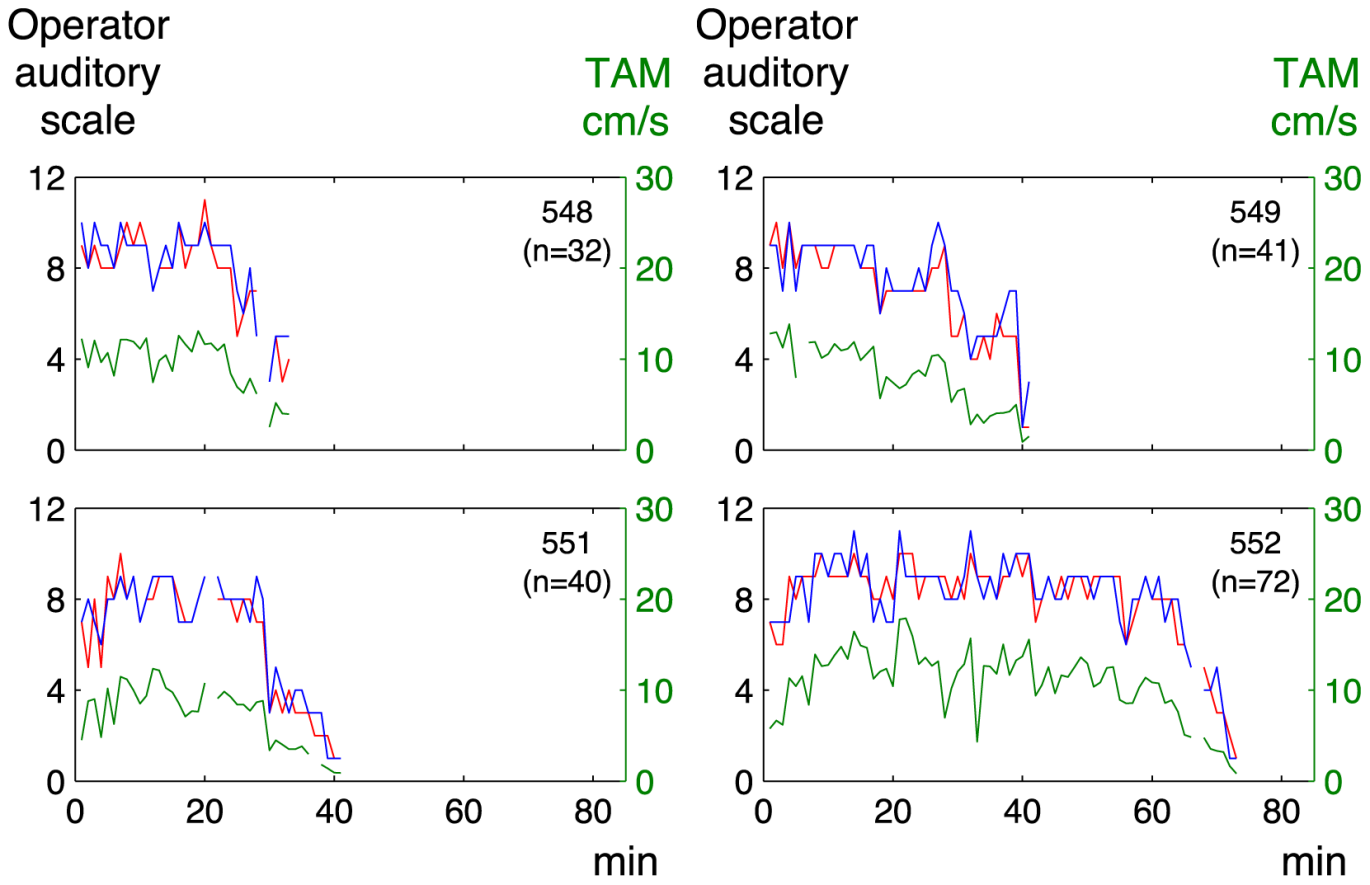
Operator auditory scale and time average mean velocity for individual Doppler recordings in the anemia model. For explanations see the legend of figure 6.

A negative correlation was found between the operator’s classification of sound (auditory scale) and PI. In the embolization model, median Rho was −0.73 (range of the coefficients for individual lamb fetuses −0.35– −0.94) (Table 1) and in the anemia model, the corresponding median Rho was −0.68 (range −0.57– −0.78) (Table 2). There was a positive correlation between auditory scale and TAM, median Rho 0.80 (range 0.58–0.95) and median Rho 0.90 (range 0.78–0.95) in the embolization and anemia model, respectively. A still stronger correlation was found between TAM and the results of sound spectrum analyses. In the embolization model, for MAX_peak_ the median r was 0.91 (range 0.88–0.97) and for MAX_peak-15 dB_, 0.91 (range 0.82–0.98). In the anemia model, the corresponding r values were 0.92 (range 0.78–0.96) and 0.96 (range 0.89–0.98), respectively.

**Table 1:** Correlation between operator auditory scale, Doppler spectrum parameters and sound spectrum analysis of umbilical artery Doppler recordings in the fetal lamb embolization model.

	Doppler spectrum		Sound spectrum	
	TAM	PI	MAX_peak_	MAX_peak-15 dB_
Operator	0.80	−0.73	0.77	0.64
auditoryscale*	(0.58–0.95)	(−0.35–0.94)	(0.50–0.94)	(0.48–0.86)
TAM**		−0.77	0.91	0.91
		(−0.72–0.89)	(0.88–0.97)	(0.82–0.98)
PI**			−0.79	−0.65
			(−0.67–0.90)	(−0.34–0.85)
MAX_peak_ **				0.84
				(0.64–0.93)

The values are medians (ranges) of the individual correlation coefficients. TAM: time average mean velocity; PI: pulsatility index; MAX_peak_: sound frequency with maximum energy content; MAX_peak-15 dB_: frequency band at maximum energy level minus 15 dB.

* Spearman’s rank correlation coefficient (Rho);

** Pearson’s correlation coefficient (r).

**Table 2:** Correlation between operator auditory scale, Doppler spectrum parameters and sound spectrum analysis of umbilical artery Doppler recordings in the fetal lamb anemia model.

	Doppler spectrum		Sound spectrum	
	TAM	PI	MAX_peak_	MAX_peak-15 dB_
Operator	0.90	−0.68	0.85	0.91
auditoryscale*	(0.78–0.95)	(−0.57–0.78)	(0.70–0.93)	(0.80–0.94)
TAM**		−0.79	0.92	0.96
		(−0.72–0.90)	(0.78–0.96)	(0.89–0.98)
PI**			−0.67	−0.85
			(−0.56–0.84)	(−0.71–0.87)
MAX_peak_ **				0.93
				(0.69–0.94)

The values are medians (ranges) of the individual correlation coefficients. TAM: time average mean velocity; PI: pulsatility index; MAX_peak_: sound frequency with maximum energy content; MAX_peak-15 dB_: frequency band at maximum energy level minus 15 dB.

* Spearman’s rank correlation coefficient (Rho);

** Pearson’s correlation coefficient (r).

Figures **4** **and** **5** illustrate the relation between the operator auditory analysis and PI in individual lamb fetuses for the embolization and anemia models, respectively. The corresponding relation between the auditory analysis and TAM is shown in figures **6** **and** **7**.

**Figure 8:**
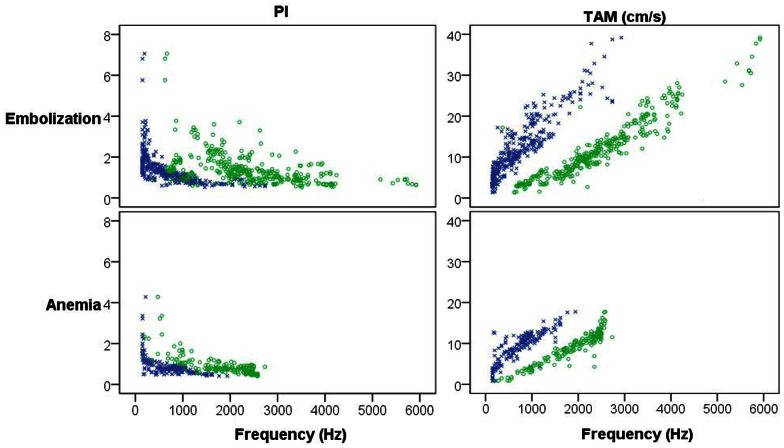
Sound spectrum analysis in the embolization and anemia models, respectively. PI: pulsatility index; TAM: time average mean velocity; blue symbols: maximum energy content (MAX_peak_); green symbols: frequency band at maximum energy level minus 15 dB (MAX_peak-15 dB_).

The results of the sound spectrum analysis in the two models are presented in figure **8**, where MAX_peak_ and MAX_peak-15 dB_ are shown in relation to PI and TAM for the embolization and anemia models, respectively.

## Discussion

We found that the subjectively perceived timbre of Doppler sound signals significantly correlated both with PI and TAM-velocity, the correlation for the latter variable being stronger. In clinical obstetrics, PI, calculated from the envelope of blood velocity Doppler signals recorded from umbilical artery, is a well-established tool for surveillance of high-risk pregnancies. However, this index only provides indirect information on the hemodynamics and resistance to flow in the placenta. A measure more closely associated to umbilical blood flow might provide valuable additional information. In our study, using an analysis of the sound spectrum, we have developed an objective measure characterizing the sound signals. The results of the sound analysis correlated significantly with the results of subjective audio classification by an experienced sonographer.

Two different lamb models have been used to establish various degrees of pathological flow velocity waveforms, similar to those seen in clinical examinations of complicated pregnancies. The embolization model was designed to mimic the situation in pregnancies with intrauterine growth restriction caused by placental pathology giving increased resistance to blood flow in umbilical arteries [9]–[12]. The fetal anemia model attempted to reproduce hyperdynamic fetal circulation in fetuses with low hematocrit, e.g. in cases of isoimmunization or parvovirus B19 infection [13]. Previously, in non-hydropic fetuses of isoimmunized pregnancies, a significant positive correlation was reported between the deviation in hemoglobin concentration and in TAM recorded from the fetal descending aorta [14]. In our experimental study, unambiguous results of the sound frequency analyses have been seen in both models. However, as the changes in umbilical artery flow velocity waveforms have been induced as an acute procedure in the lamb models, this might not necessarily reflect the changes in human pregnancies, where the underlying pathological mechanisms develop chronically. Nevertheless, the perceived timbre of Doppler audio signals in our experimental study resembled very closely to that perceived by the operator in clinical situations.

The results of Doppler examinations of umbilical artery flow velocity in human pregnancies are dependent on the quality of the primary signals. In order to achieve optimal quality and reproducibility it is important to use a standardized examination protocol and to avoid possible errors due to technical and/or biological pitfalls. Some of the most common errors and pitfalls in human examinations, e.g. fetal breathing movements and fetal general movements were minimized as the lamb fetuses were under general anesthesia. Uterine contractions might affect the placental circulation, however, the pregnant ewe has a flaccid uterus and, during the experiments, no discernible contractions were noticed.

Based on the clinical experience that Doppler audio signals recorded from the umbilical artery can vary between fetuses, in spite of no obvious differences in PI, we have tried to find an equivalent to the subjective auditory perception. The best correlation was found between the auditory classification and TAM-velocity. TAM**, i.e. time-averaged weighted mean,** reflects the blood flow as it is based on the total content of the Doppler shift spectrum as captured by the ultrasound equipment and is used for calculation of volume flow [15], [16]. The volume flow estimation is hampered by errors both in vessel diameter measurements and in the estimation of TAM [17]. TAM in itself has not been used clinically to characterize the fetoplacental circulation, probably because of its instability and dependence on acquisition circumstances [18].

The operator auditory classification of the Doppler audio signals demonstrated a strong correlation to TAM-velocity and also to the results of sound spectrum analyses (MAX_peak_ and MAX_peak-15 dB_). There was also a highly significant correlation between the TAM and the sound spectrum parameters, suggesting that the operator listens to spectral characteristics in the audio signals that are captured by the two objective analysis methods. In the sound spectrum analysis, two prominent features related to auditory perception of sounds were identified – a frequency band with the maximum energy content and a band with the high frequency cut-off. These spectrum features were selected based on previous studies suggesting that we humans listen to spectral peaks in a continuous signal such as speech [8], [19] and when these peaks are missing in the signals (e.g. low-pass filtered noise) we listen to the high frequency cut-off [20]. In the present study, an equivocal cut-off frequency could not be identified in all recordings and, hence, we selected a more stable cut-off frequency (i.e., the MAX_peak-15 dB_) that still is closely related to the perception of the cut-off frequency. The sound spectrum analysis was made using at least two (mostly more than 8–10) complete heart cycles. The use of several heart cycles increases the robustness of the method in comparison to any analysis made on single selected cycles which is the case when estimating the TAM.

Processing data containing frequencies has to be done carefully with thoughtfulness considering some prerequisite, e.g. that the insonation angle is as low as possible. The Doppler shift is also dependent on the center frequency of the ultrasound pulse. Therefore, the choice of transducer and its transmitting frequency will affect the perceived sound. For a general use of MAX_peak_ and MAX_peak-15 dB,_ these measures need to be normalized according to the transmitted ultrasound frequency.

In summary, we have found in animal models that audio spectrum analysis reflects the subjective perception of Doppler sound signals and the TAM-velocity estimated in the Doppler shift spectrum recorded from the umbilical artery. Whether or not the two new parameters (MAX_peak_ and MAX_peak-15 dB_) can improve the predictive value of Doppler examinations in high-risk pregnancies should be evaluated in prospective clinical studies.

## References

[1] Maršál K (2009) Obstetric management of intrauterine growth restriction. Best Pract Res Clin Obstet Gynecol 23: 857–870. 10.1016/j.bpobgyn.2009.08.011 19854682

[2] Gosling RG, Dunbar G, King DH, Newman DL, Side CD, et al (1971) The quantitative analysis of occlusive peripheral arterial disease by a non-intrusive ultrasonic technique. Angiology 22: 52–55. 5101050 10.1177/000331977102200109

[3] Gudmundsson S, Maršál K (1988) Umbilical artery and uteroplacental blood flow velocity waveforms in normal pregnancy – A cross-sectional study. Acta Obstet Gynecol Scand 67: 347–354. 3051883

[4] Welch P (1967) The use of fast Fourier transform for the estimation of power spectra: A method based on time averaging over short, modified periodograms. IEEE Trans Audio Electroacoust AU-15: 70–73.

[5] Jensen J (1996) Estimation of blood velocities using ultrasound. Cambridge: Cambridge University Press. 178 p.

[6] Reference manual: HDI 5000 ultrasound system (2000) ATL ultrasound, Bothell, WA: Philips Medical Systems 4703-0027-03 Rev A. p.15–9.

[7] Moraes R, Aydin N, Evans DH (1995) The performance of three maximum frequency envelope detection algorithms for Doppler signals. J Vasc Invest 1: 126–134.

[8] Alcantara JI, Holube I, Moore BC (1996) Effects of phase and level on vowel identification: data and predictions based on a nonlinear basilar-membrane model. J Acoust Soc Am 100: 2382–2392. 8865645 10.1121/1.417948

[9] Morrow R, Adamsson L, Bull S, Ritchie K (1989) Effect of placental embolization on the umbilical artery velocity waveform in the fetal sheep. Am J Obstet Gynecol 161 4: 1055–1060. 10.1016/0002-9378(89)90783-7 2679101

[10] Thompson R, Trudinger B (1990) Doppler waveform pulsatility index and resistance, pressure and flow in the umbilical placental circulation: an investigation using a mathematical model. Ultrasound Med Biol 16: 449–458. 2238251 10.1016/0301-5629(90)90167-b

[11] Trudinger B, Giles W, Cook C, Bombardieri J, Collins L (1985) Fetal umbilical artery flow velocity waveforms and placental resistance: clinical significance. Br J Obstet Gynaecol 92: 23–30. 4038455 10.1111/j.1471-0528.1985.tb01044.x

[12] Madazli R, Somunkiran A, Calay Z, Ilvan S, Aksu F (2003) Histomorphology of the placenta and the placental bed of growth restricted foetuses and correlation with Doppler velocimetries of the uterine and umbilical arteries. Placenta 24: 510–516. 12744927 10.1053/plac.2002.0945

[13] Mari G (2000) Noninvasive diagnosis by Doppler ultrasonography of fetal anemia due to maternal red-cell alloimmunization. New Engl J Med 342: 9–14. 10620643 10.1056/NEJM200001063420102

[14] Nicolaides KH, Bilardo CM, Campbell S (1990) Prediction of fetal anemia by measurement of the mean blood velocity in the fetal aorta. Am J Obstet Gynecol 162: 209–212. 2105648 10.1016/0002-9378(90)90852-x

[15] Eik-Nes S, Maršál K, Brubakk A, Kristofferson K, Ulstein M (1982) Ultrasonic measurement of human fetal blood flow. J Biomed Engng 4: 28–36. 7078139 10.1016/0141-5425(82)90023-1

[16] Acharya G, Wilsgaard T, Berntsen GKR, Maltau JM, Kiserud T (2005) Doppler-derived umbilical artery absolute velocities and their relationship to fetoplacental volume blood flow: a longitudinal study. Ultrasound Obstet Gynecol 25: 444–453. 15816007 10.1002/uog.1880

[17] Eik-Nes S, Maršál K, Kristoffersen K (1984) Methodology and basic problems related to blood flow studies in the human fetus. Ultrasound Med Biol 10: 329–337. 6235656 10.1016/0301-5629(84)90167-4

[18] Thompson RS, Trudinger BJ, Cook CM (1986) A comparison of Doppler ultrasound waveform indices in the umbilical artery – II Indices derived from the mean velocity and first moment waveforms. Ultrasound Med Biol 11: 845–854. 10.1016/0301-5629(86)90002-5 2949413

[19] Diehl RL, Lotto AJ, Holt LL (2004) Speech perception. Annu Rev Psychol 55: 149–179. 14744213 10.1146/annurev.psych.55.090902.142028

[20] Fastl H, Zwicker E (2007) Psychoacoustics (2^nd^ ed). Berlin: Springer Verlag.

